#  Neonatal Adrenal Hemorrhage presenting as Prolonged Hyperbilirubinemia 

**Published:** 2016-04-10

**Authors:** Jasbir Singh, Poonam Dalal, Kapil Bhalla, Kamal Nain Rattan

**Affiliations:** 1 Department of Pediatrics, PGIMS, Rohtak, Haryana India 124001; 2 Department of Pediatric Surgery, PGIMS, Rohtak, Haryana India 124001

**Dear Sir**

Adrenal hemorrhage is quiet uncommon in neonates with reported incidence of 0.2%-0.55% [1]. In neonates, adrenal glands are large in size and have abundant vascular supply, so more prone to hemorrhage. Clinical manifestations may vary from relatively benign as anemia, flank mass, discoloration of scrotum, neonatal jaundice to life threatening addisonian crisis depending upon the extent of hemorrhage [1]. Incidences of hemorrhage are more after prolonged labor, birth trauma, birth asphyxia, large for dates babies, sepsis and coagulation disorders [2]. We are describing a case of adrenal hemorrhage who presented with prolonged jaundice.

A full term 3.2 Kg male baby was born by vacuum assisted vaginal delivery to a primigravida mother. The baby had history of birth asphyxia with one and 5 minutes APGAR score of 4 and 7, respectively. The patient was discharged home after 48hrs of life on breast feed. Baby was readmitted for neonatal jaundice on day 8th of life. Total serum bilirubin (TSB) was 17.5 mg/dl with direct component of 0.7 mg/dl. Baby was transfused blood in view of anemia. Patient was referred to our tertiary care centre on 13th day of life in view of persistent hyperbilirubinemia despite intensive phototherapy.

On examination patient was icteric up to sole and had a small cephalhematoma at left parietal region. A mass was palpable in left lumber region with ill-defined margins. Initial investigations revealed hemoglobin 14 g/dl, TSB 19.1 mg/dl with direct component of 0.6 mg/dl and random blood sugar was 76mg/dl. Blood groups of both mother and baby were B positive. Direct Coombs test was negative, reticulocyte count was 3.0%, G6PD levels, thyroid profile and coagulation profile were within normal limits. Sepsis screen and TORCH profile was negative. Kidney functions were normal but liver functions were deranged with SGOT 206 U/L, SGPT 86 U/L, Serum proteins 5.0 g/L with A/G ratio of 1.6. Urine analysis for reducing substances and bile pigments was negative. Baby was passing pigmented stools. On ultrasonography (USG), a large hypo echoic lesion of size 7.1 cm× 4.6 cm was seen at upper pole of left kidney suggestive of adrenal hemorrhage. Computed tomography of abdomen confirmed the diagnosis of adrenal hemorrhage (Fig. 1). Serum bilirubin levels started decreasing and became 12.2 mg/dl so phototherapy was stopped after one week. There was no rebound hyperbilirubinemia. Patient was discharged after another week in stable condition. Adrenal hemorrhage was monitored regularly by USG abdomen and it decreased in size gradually over a period of next 8 weeks. 

Neonatal jaundice is said to be prolonged when visible jaundice persist beyond 14 days in term and 21 days of life in preterm. Commonly reported etiological factors are hemolytic diseases, breast milk jaundice, hypothyroidism, infections, Crigler-Najjar syndrome and extravascular blood [3]. Adrenal hemorrhage is very rarely reported as a cause for prolonged jaundice with isolated case reports in literature [1, 2]. The index case had both cephalhematoma and adrenal hemorrhage, but cephalhematoma was very small. Prolonged labor leading to birth asphyxia was the underlying cause in the index case. Adrenal hemorrhage may remain asymptomatic or may manifest as life threatening addisonian crisis. On the other hand clinical presentation depends upon size of hemorrhage and area of adrenal cortex involved. Hemorrhage is more common on right side adrenal gland. 

Ultrasonography is the investigation of choice to diagnose adrenal hemorrhage and to follow up in neonatal period [5]. Neuroblastoma has to be differentiated from adrenal hemorrhage. In the index case normal VMA levels and absence of calcifications with no contrast enhancement on CECT abdomen excluded possibility of neuroblastoma. Serial USG monitoring is method of choice during conservative management. Adrenal hemorrhage usually takes 3 weeks to 6 months to resolve completely [4, 5]. In index case, adrenal hemorrhage resolved in 12 weeks after birth.

**Figure:**
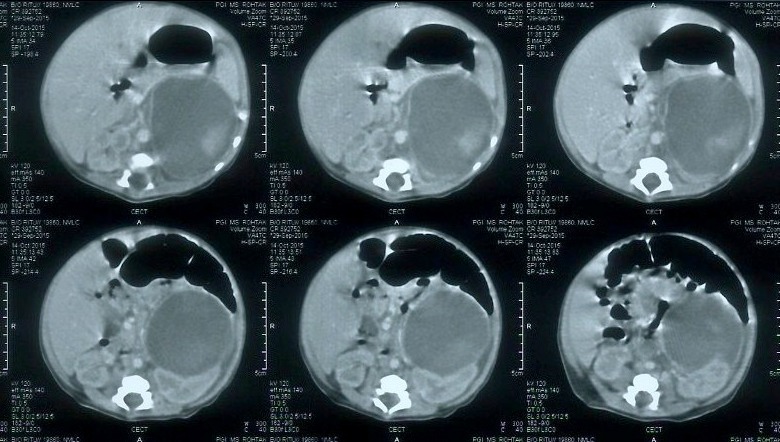
Figure 1: Computed Tomography abdomen of neonate showing large adrenal hemorrhage on left side.

## Footnotes

**Source of Support:** Nil

**Conflict of Interest:** None
